# FD^2^-YOLO: A Frequency-Domain Dual-Stream Network Based on YOLO for Crack Detection

**DOI:** 10.3390/s25113427

**Published:** 2025-05-29

**Authors:** Junwen Zhu, Jinbao Sheng, Qian Cai

**Affiliations:** 1 Nanjing Hydraulic Research Institute, Nanjing 210029, China; jwzhu@nhri.cn (J.Z.); wizertize@foxmail.com (Q.C.); 2 Country Dam Safety Management Center, The Ministry of Water Resources, Nanjing 210029, China

**Keywords:** crack detection, FD^2^-YOLO, dual backbone, DIFF, DIA Head

## Abstract

Crack detection in cement infrastructure is imperative to ensure its structural integrity and public safety. However, most existing methods use multi-scale and attention mechanisms to improve on a single backbone, and this single backbone network is often ineffective in detecting slender or variable cracks in complex scenarios. We propose a novel network, FD^2^-YOLO, based on frequency-domain dual-stream YOLO, for accurate and efficient detection of cement cracks. Firstly, the model employs a dual backbone architecture, integrating edge and texture features in the frequency domain with semantic features in the spatial domain, to enhance the extraction of crack-related features. Furthermore, the Dynamic Inter-Domain Feature Fusion module (DIFF) is introduced, which uses large-kernel deep convolution and Hadamard to enable the adaptive fusion of features from different domains, thus addressing the problem of difficult feature fusion due to domain differences. Finally, the DIA-Head module has been proposed, which dynamically focuses on the texture and geometric deformation features of cracks by introducing the Deformable Interactive Attention Module (DIA Module) in Decoupled Head and utilizing its Deformable Interactive Attention. Extensive experiments on the RDD2022 dataset demonstrate that FD^2^-YOLO achieves state-of-the-art performance. Compared with existing YOLO-based models, it improves mAP50 by 1.3%, mAP50-95 by 1.1%, recall by 1.8%, and precision by 0.5%, validating its effectiveness in real-world object detection scenarios. In addition, evaluation on the UAV-PDD2023 dataset further confirms the robustness and generalization of our approach, where FD^2^-YOLO achieves a mAP50 of 67.9%, mAP50-95 of 35.9%, recall of 61.2%, and precision of 75.9%, consistently outperforming existing lightweight and Transformer-based detectors under more complex aerial imaging conditions.

## 1. Introduction

Cement is a fundamental material in national infrastructure and is widely utilized in diverse engineering projects such as road construction, bridges, and dams. However, due to long-term exposure to harsh environmental conditions, such as rainfall, temperature fluctuations, and chemical erosion, cement surfaces are prone to damage and cracking. If not detected and repaired promptly, these cracks may propagate, potentially leading to severe structural degradation and, in extreme cases, catastrophic failures. At present, conventional crack-detection techniques principally comprise manual inspection and equipment detection. Manual inspection depends on visual experience for the observation and documentation of cracks. This method is characterized by inefficiency, strong subjectivity, lack of precision, and poor safety. In contrast, detection equipment based on ultrasonic [1], infrared imaging [2], ground-penetrating radar [3], and other technologies can locate cracks. However, these methods are also limited by high equipment requirements, high cost, complex calculations, and the inability to identify and classify cracks, which hinders their practical application. Consequently, there is an urgent need to develop efficient and accurate automatic crack-detection methods, which are of great significance for ensuring structural safety, prolonging the life of facilities, and promoting the development of engineering maintenance technology.

The advent of information technology and image recognition has engendered novel methodologies for automated crack detection. Initially, the primary focus of computer vision techniques was on conventional image processing and traditional machine learning algorithms. Methods such as Threshold Segmentation [4], Edge Detection [5], and Minimal Path Selection (MPS) [6] detect cracks by exploiting intensity differences between the cracks and background. However, these approaches frequently encounter challenges in real-world scenarios due to their vulnerability to noise, lighting variations, and background clutter. To address these limitations, researchers have proposed machine learning methods that offer enhanced adaptability and classification accuracy. Examples of such methods include algorithms based on K-Nearest Neighbors (KNN) [7], Support Vector Machines (SVM) [8], and Random Forest (RF) [9]. Shi et al. [10] proposed a road crack-detection framework that leverages Random Forest classifiers enriched with integral channel features, while Ai et al. [11] developed a pixel-level detection method using multi-scale neighborhood information and pixel gray values. Despite the performance enhancements, these models are heavily dependent on manual feature engineering and extensive data preprocessing, which limits their scalability and effectiveness in complex, high-dimensional environments.

In recent years, deep learning-based methods have revolutionized the field of concrete crack detection by replacing handcrafted features with end-to-end feature learning. These models demonstrate superior generalization and localization accuracy, especially in complex scenarios. In the domain of deep learning, crack-detection methodologies are predominantly categorized into two primary approaches: object detection and semantic segmentation. Segmentation-based methods achieve fine-grained detection through pixel-level prediction. For instance, Li et al. [12] proposed Mini-Unet architecture by replacing standard convolutional layers with Depthwise Separable Convolutions (DSConv) to enable efficient tunnel crack segmentation. Li et al. [13] enhanced the performance of SegFormer in fine crack detection by integrating Cross-Entropy (CE) and Dice loss functions. Zhu et al. [14] developed RHACrackNet, a lightweight network incorporating hybrid attention blocks (HABs) and residual blocks, achieving an F1 score of 94.94% on a self-built dataset of 789 pavement crack images. Mayya et al. [15] propose a triple-stage framework combining YOLO ensemble for crack detection, MobileNetV2U—a net for segmentation, and spectral clustering for crack pattern classification to improve the accuracy of stonemasonry crack identification. Zheng et al. [16] introduced the CAL-ISM framework integrating semi-supervised active learning and stacked convolutional autoencoder (SCAE) features, achieving full-process automation from crack identification to width measurement through an eight-directional region-growing algorithm. However, the current segmentation’s performance falls short of meeting the requirements for practical applications. Compared to other tasks, segmentation is inherently more complex and challenging to optimize. Moreover, acquiring large-scale segmentation datasets is difficult, leading most existing studies to rely on limited sample sizes [17]. Additionally, complex pavement interferences (oil stains, shadows, repairs) often induce segmentation artifacts requiring post-processing noise suppression.

To balance detection accuracy with computational efficiency, many researchers have proposed innovations within single-stage object detection frameworks. For example, Raushan et al. [18] created a dataset of 3750 real images with multi-feature backgrounds and trained YOLOv3 to v10 models for damage detection in concrete structures. They evaluated the models’ performance under three scenarios and found that YOLOv4 achieved the best results, with a precision of 92.2%, a recall of 86.8%, and an F1 score of 88.9%. Li et al. [17] developed a grid-based classification and box-based detection fusion model (GCBD) based on YOLO v5, improved accuracy and efficiency through a shared backbone network and multi-task joint training, proposed the NMS-ARS algorithm for post-processing optimization, and validated it on a large-scale double-labeled dataset to achieve the automatic identification of asphalt pavement cracks. Dong et al. [19] enhanced YOLOv5-AH with CBAM attention modules and quad-scale prediction heads, enabling real-time 72 FPS detection in mobile robot-acquired complex scenarios. Zhao et al. [20] proposed the MED-YOLOv8s model, which integrates MobileNetV3 and Depthwise Separable Convolutions, along with an Efficient Channel Attention (ECA) mechanism to optimize inter-channel relationships while maintaining low computational cost. He et al. [21] introduced the LSF-RDD model, which employs a multi-scale feature fusion strategy and incorporates a specialized SDB-Loss function to enhance localization sensitivity under complex damage conditions. Similarly, Zeng et al. [22] developed YOLOv8-ES, incorporating dynamic convolution and hybrid attention to better capture slender crack structures, along with an adaptive loss function to improve robustness against low-quality samples. Dong et al. [23] proposed YOLOv8-CD, optimized explicitly for concrete cracks through the use of large-kernel attention modules and lightweight convolutions, achieving a strong balance between accuracy and efficiency. Further advancements include the EF-RT-DETR model by Han et al. [24], which combines re-parameterized convolution and multi-scale focus modules to reduce model size without compromising detail preservation. Ding et al. [25] presented SCD-YOLO, integrating cross-scale attention with the SWC2f module to expand the receptive field and employing the CCAFM and DyHead modules for enhanced recognition of complex crack morphologies. Li et al. [26] developed LHA-Net, a lightweight yet expressive network incorporating global (DGFM) and local (HLFM) feature modules, fused using the AWAM mechanism. Youwai et al. [27] proposed YOLO9tr, introducing a PSA attention block in the neck to boost critical feature extraction across various cement damage types. Zhou et al. [28] designed a hybrid attention module that performs spatial bidirectional feature aggregation, embedding channel and positional information to capture long-range dependencies of crack patterns. Chen et al. [29] introduced a multi-scale mobile attention network that leverages deep separable convolutions and hybrid attention mechanisms to enhance pavement crack segmentation accuracy by modeling spatial and channel relationships. Zhang et al. [30] proposed a cross-layer transfer network, using multi-scale cross-layer fusion to address the problem of weak signals in tiny crack detection by ensuring progressive information transmission across feature layers.

It is important to note that, given the inherent problems of limited sensory field and information loss during pooling in CNN structure, researchers began to explore the combination of transformer and CNN. He et al. [31] enhanced the detection of road surface cracks by integrating transformer blocks into convolutional neural networks to expand the receptive field. They also introduce a novel AFM model to enhance the acquisition and integration capabilities of high-level and low-level feature maps. Zhang et al. [32] constructed a C-Transformer model that combines convolutional operations and a self-attention mechanism, achieving excellent performance in the task of asphalt pavement crack detection. Zhang et al. [33] further designed a parallel convolutional–transformer hybrid module with embedded fusion units, which contains embedded fusion units. The transformer hybrid module can simultaneously capture complex local features and global contextual information, and effectively integrate contextual information at different scales through specific fusion units while preserving the texture details of the cracks. Wang et al. [34] put forward a concrete crack-detection network based on a transformer architecture with receptive field attention and adaptive loss. Wan et al. [35] propose BR-DETR, a novel bridge damage detection model based on detection transformers, which integrates deformable convolutions, convolutional project attention, and locally enhanced feed-forward layers, achieving superior performance on augmented bridge damage datasets.

Notwithstanding these advances, prevailing methodologies principally emphasize multi-scale representation and attention mechanisms. However, several key limitations persist. Firstly, single-backbone networks often struggle to effectively extract slender or reticulated cracks, resulting in the loss of fine-grained spatial details. Secondly, spatial-domain features are highly susceptible to background interference, which hampers the detection of low-contrast textures. Thirdly, both standard and dynamic convolutional operations are inherently limited in capturing irregular crack morphologies, such as bifurcations and web-like structures. These challenges underscore a critical gap in the development of robust and generalizable crack-detection frameworks, especially under diverse and complex environmental conditions.

To address the limitations identified, a novel frequency-domain dual-stream network (FD^2^-YOLO) is proposed for robust cement crack detection. The key contributions of this work are as follows:

1. Dual-Domain Collaborative Feature Extraction Architecture: We design a parallel dual-stream backbone comprising a spatial-domain branch and a frequency-domain branch. The spatial branch consists of multi-layer CNN modules to capture global semantic information, while the frequency branch introduces the C3K2-LWN module, which utilizes wavelet decomposition to enhance edge and texture representations. The coordinated fusion of spatial and frequency features significantly improves the accuracy of detecting slender cracks under complex backgrounds.
2. Dynamic Inter-Domain Feature Fusion Module: We construct a dynamic inter-domain feature integration module (DIFF) that combines large-kernel Depthwise Convolution with a Hadamard product-based attention mechanism. This design enables channel-wise importance modeling, facilitating the adaptive integration of spatial-domain contextual information and frequency-domain edge details. The module effectively mitigates domain discrepancy in feature distribution, enhancing the network’s ability to represent and discriminate complex crack morphologies.
3. Deformable Interactive Attention Detection Head: Building upon the Decoupled Head paradigm, we incorporate a DIA Module, which combines multi-scale pooling and dynamic modulation mechanisms. This detection head is capable of accurately perceiving grayscale, texture, and localized deformation features of cracks, thereby improving precision in localization and classification under challenging conditions.

Accordingly, this study aims to solve the following scientific problem: how to construct an efficient and generalizable detection framework capable of accurately identifying complex crack morphologies, including slender, low-contrast, and bifurcated structures, across varying environments. The scientific novelty of this work lies in the integration of frequency-domain analysis with spatial-domain modeling in a dual-backbone YOLO framework, the application of wavelet-enhanced feature encoding, and the DIFF module for adaptive inter-domain fusion. From a practical standpoint, it offers a scalable solution for automated crack inspection in cement-based infrastructure, with broad implications for civil safety monitoring, preventative maintenance, and structural health assessment.

## 2. Methods

### 2.1. Research and Development Process of FD^2^-YOLO

**Figure 1:**
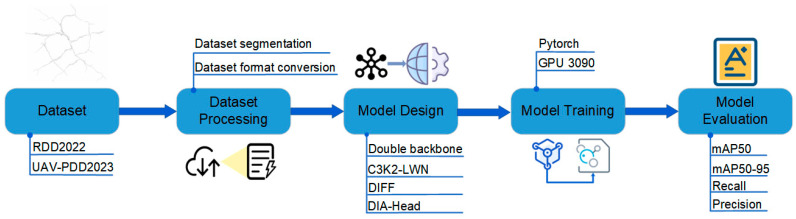
The FD^2^-YOLO study phase is delineated by a flowchart.

The proposed crack-detection system follows a structured five-stage workflow, as illustrated in Figure 1. It begins with the data preparation stage, where images from the RDD2022 benchmark dataset are stratified into training (70%), validation (20%), and testing (10%) subsets. These images are then converted into YOLO format. Next, the system proceeds to feature extraction using the FD^2^-YOLO architecture, which adopts a dual-backbone design. The spatial-domain backbone employs C3K2 residual blocks to capture local spatial details, while the frequency-domain backbone integrates C3K2-LWN wavelet modules to extract frequency-aware features. These complementary features are then merged through the DIFF module (Dynamic Inter-Domain Feature Fusion), enabling adaptive integration of spatial and frequency-domain information. The fused representations are passed to the DIA Head (Deformable Interactive Attention Head), which enhances detection performance through deformable convolutions and attention mechanisms. In the final training and evaluation stage, the model is optimized using PyTorch 1.12 on an NVIDIA RTX 3090 GPU, Santa Clara, CA, USA. The effectiveness of the proposed system is verified across multiple evaluation metrics, including mAP@50, mAP@50-95, recall, and precision, all of which demonstrate strong and reliable performance in crack-detection tasks.

### 2.2. The FD^2^-YOLO Model Structure

The latest iteration of Ultralytics’ target detection model, designated as YOLOv11 [36], has been developed to achieve highly accurate and efficient real-time detection. In comparison with previous iterations of the YOLO series, YOLOv11 has undergone substantial optimization of model architecture and training strategy. Its architecture is composed of three constituent parts: The Backbone, Neck, and Head components. The Backbone part is based on DarkNet53 and uses the C3K2 module instead of the traditional C3 structure, thus enhancing the feature extraction capability. The Neck part introduces an enhanced feature fusion module, which combines the Feature Pyramid Network and Path Aggregation Network to improve information utilization efficiency. Finally, the Head part is responsible for accurately detecting targets of varying sizes, facilitated by multi-scale feature maps.

**Figure 2:**
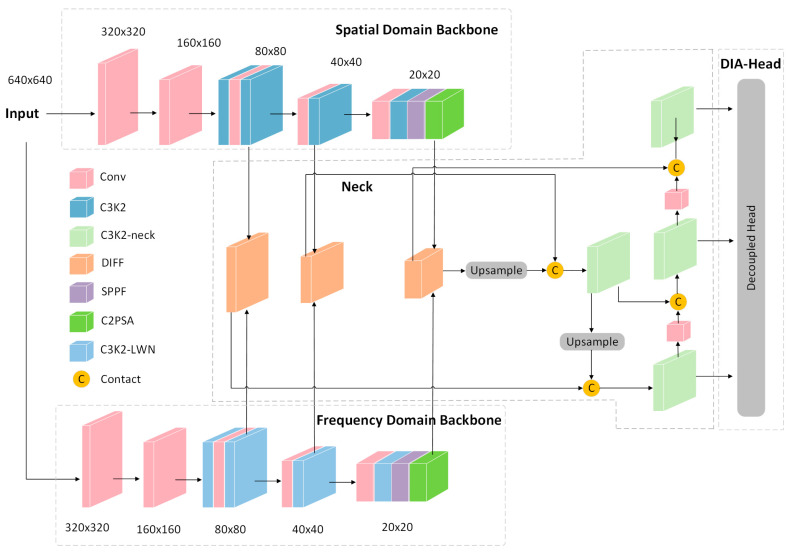
FD^2^-YOLO model structure diagram.

The FD^2^-YOLO model is proposed as a means of enhancing the accuracy of crack detection. As demonstrated in Figure 2, the model incorporates a Dual-Domain Collaborative Feature Extraction Architecture. The model is fed a 640 × 640 crack image with three channels (RGB), which simultaneously input into two parallel backbones:

The spatial-domain backbone (top-left branch) retains the convolutional architecture of YOLOv11, employing a sequence of convolutional layers, C3K2 modules, and SPPF blocks to progressively downsample the input and extract high-level semantic features. At each stage, the spatial resolution is reduced by a factor of two (e.g., from 640 × 640 to 320 × 320, then to 160 × 160, and 80 × 80). Concurrently, the frequency-domain backbone (bottom-left branch) incorporates the C3K2-LWN module, which performs multi-scale frequency decomposition to enhance the representation of crack edges and texture features. The branch operates in the frequency domain, thereby facilitating the more effective separation of structural details from noise and background clutter. It provides complementary information that, when fused with spatial features, improves the overall discriminative capacity of the network.

The outputs from both backbones are then fused via the DIFF module (Dynamic Interaction and Fusion Framework), which is located in the center of the architecture. DIFF employs a dynamic alignment and combination of spatial and frequency features across multiple scales (e.g., 80 × 80, 40 × 40, 20 × 20) utilizing large-kernel convolutions and a Hadamard attention mechanism. This enhances the expressiveness of features in morphologically complex crack scenarios.

The fused features are then passed to the detection neck and subsequently to the DIA Head (Deformable Interaction Aggregation Head), a geometrically adaptive Decoupled Head for final prediction. The DIA Head incorporates deformable convolutional layers and multi-scale upsampling to enhance geometric adaptability and precisely localize cracks of varying shapes and scales. The detection head generates three prediction feature maps of dimensions 80 × 80, 40 × 40, and 20 × 20, incorporating multiple channels. These are responsible for object classification, bounding-box regression, and objectness scores.

### 2.3. Dual-Domain Collaborative Feature Extraction Architecture

**Figure 3:**
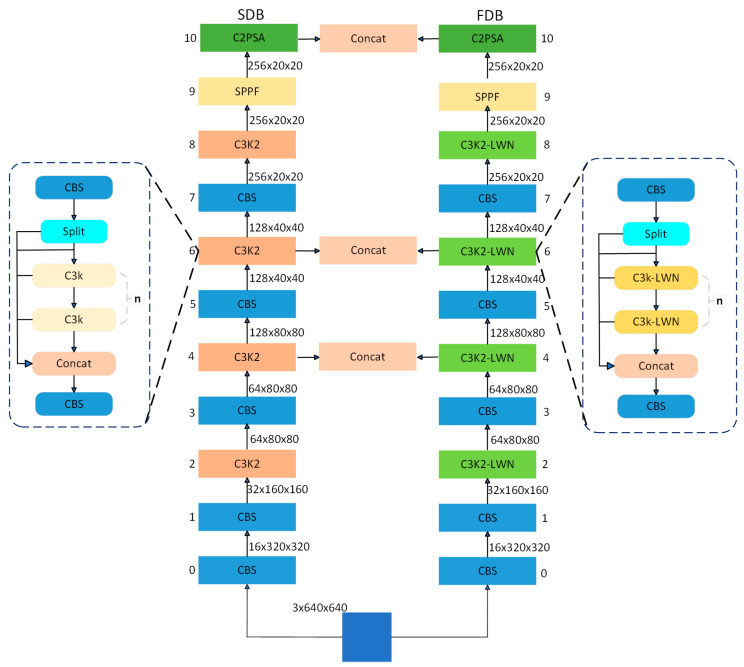
Dual-Domain Collaborative Feature Extraction Architecture.

As demonstrated in Figure 3, the Dual-Domain Collaborative Feature Extraction Architecture proposed in this paper comprises two components: the spatial-domain backbone (SDB) and the frequency-domain backbone (FDB). The function of the SDB is to capture spatial structural information, whilst the FDB is responsible for capturing frequency-domain texture features. This synergy enhances the network’s ability to perceive subtle edge variations and improves the overall feature representation. The SDB employs multi-layer gradual downsampling to continuously deepen the understanding of the semantic information of the image. Each stage comprises multiple fundamental modules, including the standard CBS (Convolution–BatchNorm–Silu) for extracting local spatial features, and the C3K2 module, which is designed based on the cross-stage residual connection (CSP) structure. The SPPF module enhances the sensory field through the multi-scale Maximal Pooling operation and extracts multi-scale contextual information. The C2PSA module introduces a parallel spatial-attention mechanism to enhance the network’s modeling ability for long-distance dependence, effectively mitigating the loss of details of features during deep-level processing.

**Figure 4:**
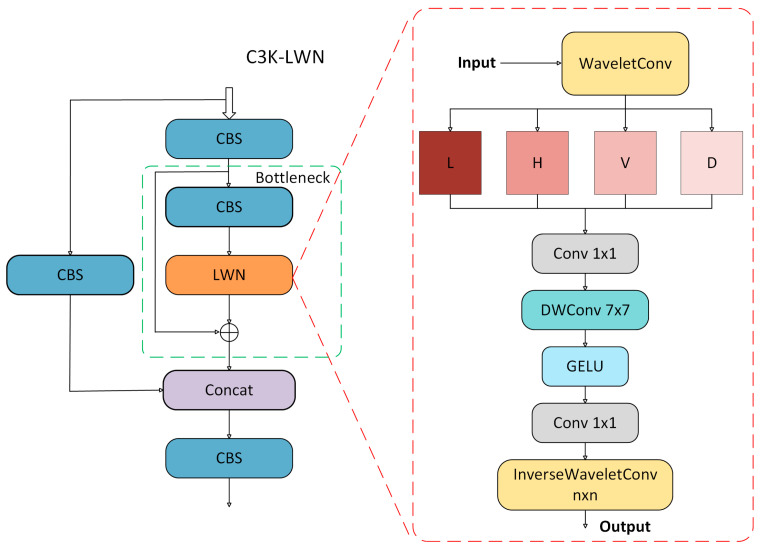
C3K-LWN structure diagram.

The FDB and SDB remain the same in structure, but the key difference is that we introduce the Learnable Discrete Wavelet Transform Node [37] (LWN) into the C3K2 module, and construct the C3K2-LWN module. Wavelet analysis excels in feature extraction compared to conventional methods. The high-frequency coefficients of this transform effectively highlight fine edges and crack boundaries. The multi-resolution nature of wavelet analysis is advantageous in that it enables the capture of both the global context and fine-grained details of an image. The sparse representation of wavelets, a key advantage, utilizes a reduced number of coefficients to retain essential information. In images of cement cracks, this property facilitates efficient encoding, emphasizing coefficients associated with crack edges and textures. This process reduces computational complexity and enhances the discriminative power of the extracted features. Furthermore, the adaptability of wavelets to irregular structures renders them superior in representing the complex and variable shapes of cracks, thus overcoming the rigid limitations of traditional CNN kernels. As shown in Figure 4, in the C3K2 module, the nested C3K module is a lightweight dual-path feature extraction structure, the core of which consists of a parallel CBS module and a Bottleneck compression layer. After the input features are initially extracted by CBS, the main path retains the original information (Shortcut), and the subpaths are compressed by the Bottleneck compression channel and stacked with the CBS module to extract the details; finally, the dual-path features are fused by Concat, which reduces the number of parameters while realizing the multi-scale feature fusion, and it is suitable for the detection of complex and variable cement crack targets. To enhance the frequency-domain sensing ability, we introduce the LWN module in Bottleneck, where wavelet decomposes the input features and extracts the low-frequency subbands (ll), horizontal (lh), vertical (hl), and diagonal (hh) high-frequency subbands, respectively. We set ${\vec{a}}_{0}$ and ${\vec{a}}_{1}$ as learnable filters.

(1) $$F_{ll}={\vec{a}}_{0}{\vec{a}}_{0}^{T},F_{lh}={\vec{a}}_{0}{\vec{a}}_{1}^{T},F_{hl}={\vec{a}}_{1}{\vec{a}}_{0}^{T},F_{hh}={\vec{a}}_{1}{\vec{a}}_{1}^{T}$$

Subsequently, all subbands are uniformly fed into a 1×1 convolution for channel compression and texture enhancement by 7×7 depth separable convolution with GELU used for the activation function, and the overall processing is expressed as:

(2) $$F_{subband}=DWConv_{7\times 7}\left(Conv_{1\times 1}\left(Decompose\left(F_{in}\right)\right)\right)$$

Finally, the reconstruction of the spatial features is achieved through an inverse transformation using InverseWaveletConv. Concurrently, the spatial feature Concat, retained by the primary path, is fused with the reconstructed spatial features. Subsequent to channel convolution by ${Conv}_{1\times 1}$, the output features are of the same size as the input. Through this joint modeling in multiple frequency domains, the C3K-LWN module effectively improves the extraction of texture, edges, and other features of cracks.

(3) $$F_{out}=Conv_{1\times 1}(Concat(CBS\left(F_{in}\right),InverseWavelet\left(F_{subband}\right)$$

### 2.4. Dynamic Inter-Domain Feature Fusion Module

**Figure 5:**
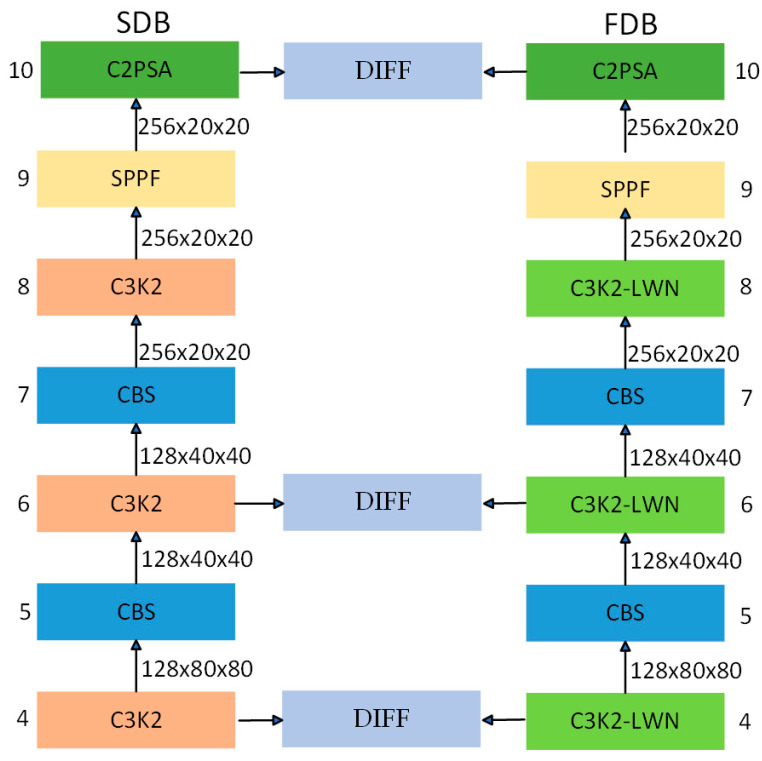
The structure of a diagram of replacing the Concat position with the Dynamic Inter-Domain Feature Fusion Module.

In the Dual-Domain Collaborative Feature Extraction Architecture, the SDB is responsible for extracting the global context information in the spatial domain, while the FDB filters the low-frequency information and highlights the high-frequency edge features through the LWN module. Each of these modules has its focus in terms of information extraction. In the event of their output features being fused directly by means of simple splicing or weighted fusion, this will result in information conflict due to the difference in feature distribution. As demonstrated in Figure 5, we propose the Dynamic Inter-Domain Feature Fusion Module (DIFF), which replaces the Concat between layers 4, 6, and 10 of the Dual-Domain Collaborative Feature Extraction Architecture. The DIFF module exploits the complementary nature of spatial- and frequency-domain features, thereby enhancing the model’s capacity to detect the target.

**Figure 6:**
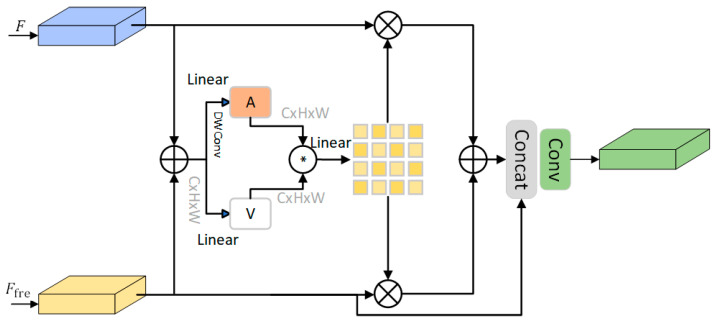
Structure of Dynamic Inter-Domain Feature Fusion Module.

As demonstrated in Figure 6, the DIFF model employs a dynamic-domain feature fusion architecture, with the left F branch input originating from the SDB, which contains abundant global semantic information, and the right $F_{fre}$ branch input stemming from the FDB, which accentuates the edge and detail information of the image. Initially, the spatial- and frequency-domain information is integrated through element-by-element summation fusion. Subsequently, the global spatial importance is computed using a large-kernel convolution, and the contribution of key features is enhanced using the Hadamard product in the fusion stage. The key feature importance is then utilized to multiply with the two branches, which fuse the learned dynamic frequency-domain features and spatial features. Finally, the features are refined through 1 × 1 convolution and Concat. The DIFF design ensures that the spatial- and frequency-domain information can be fused in a complementary way, so that the network can capture the global background information while retaining the edge and texture features effectively, thus improving the robustness and detection performance of the model.

The DIFF model is composed of three distinct stages: preliminary feature fusion, weight learning, and adaptive fusion. The purpose of the latter two stages is to ensure that spatial and frequency features can be efficiently integrated. In the preliminary feature fusion stage, the two features are initially fused using element-by-element summation. The computational formula is as follows:

(4) $$F_{sum}=F+F_{fre}$$

This addition operation enables the preliminary feature fusion without the introduction of additional parameters, thereby facilitating further interaction between the spatial and frequency features within the same feature space.

In the weight learning phase, the weights of each channel are first computed on the initial fused features using k × k Depthwise Convolution to generate channel-level modulation factors *A*. Concurrently, the modulation factors *A* and *B* are calculated using the Hadamard Product to obtain the regionally significant distribution feature map *D*. The computational formula is as follows:

(5) $$A=DWConv_{k\times k}\left(W_{1}F_{sum}\right)$$

(6) Z=A⊙V

In this context, $F_{sum}$ denotes the initial feature fusion, ${DConv}_{k\times k}$ represents the deep convolution operation, and $W_{1}$ is the linear transformation parameter.

In the adaptive fusion stage, firstly, the dynamic spatial–frequency-domain features are learned by multiplying the regionally important distribution feature map Z with F and $F_{fre}$, respectively. Consequently, the learned dynamic spatial- and frequency-domain features are summed and fused. Concurrently, in order to increase the proportion of frequency-domain features, we also combine the original $F_{fre}$ and concatenate it with the fused ones, which can well retain the detailed information in the original frequency-domain features.

(7) $$F_{out}=Conv_{1\times 1}\left(Concat\left(Z\times F+Z\times F_{fre},F_{fre}\right)\right)$$

where $F_{out}$ is the final fusion feature. This mechanism enables DIFF to dynamically adjust the feature weights of different domains according to the task requirements, thereby allowing the model to adaptively optimize the feature expression ability under different input conditions. To further enhance the fusion effect, DIFF employs a 1 × 1 convolution for feature compression, thereby rendering the fused information more compact and endowing it with enhanced discriminative ability.

### 2.5. Deformable Interactive Attention Detection Head

**Figure 7:**
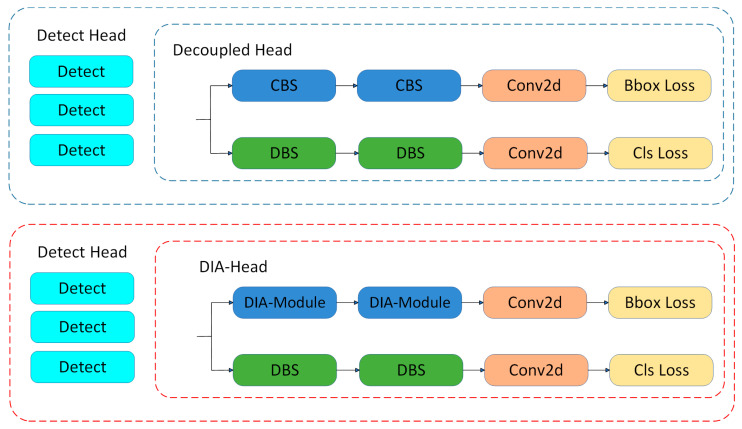
Decoupled Head and DIA-Head structure diagrams.

In the conventional YOLO Decoupled Head, as shown in Figure 7, comprising CBS and DBS (Depthwise Separable Convolution–BatchNorm–Silu) to constitute the classification and localization heads, respectively, the Decoupled Head is favored for its simplicity and high efficiency. Nevertheless, the ordinary convolution treats all regions and channels of the image equally and does not sufficiently take the uniqueness of the crack features into account. Cracks frequently exhibit elongated, irregular shapes and distinctive textures, which makes it challenging for standard convolution to concentrate on these critical features. Conventional convolution processes both the background and the crack features in the presence of a complex background, resulting in the introduction of redundant information. This has the potential to impede the precise identification of cracks and compromise the detection accuracy.

**Figure 8:**
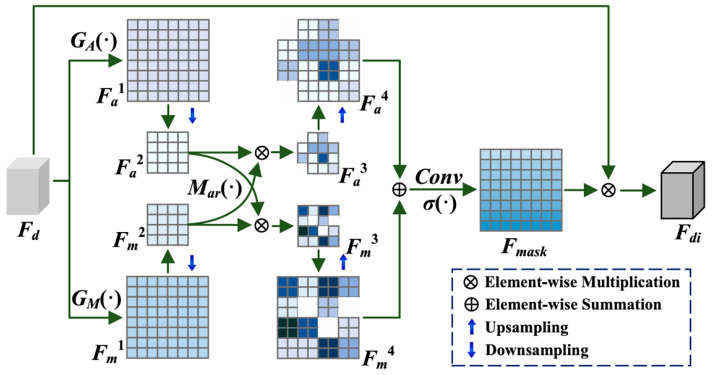
DIA-Module structure diagram.

Considering the aforementioned issues, we propose the adoption of a Deformable Interactive Attention Module [38] (DIA Module) as a replacement for the conventional convolution operation within the Decoupled Head module. As shown in Figure 8, firstly, the input crack image feature $F_{d}\in R^{W\times H\times 1}$ is processed, and $F_{a}^{1}\in R^{W\times H\times 1}$ with global smoothness and $F_{m}^{1}\in R^{W\times H\times 1}$ highlighting the local texture details are obtained by the Global Average Pooling (GA) and the Maximal Pooling (GM) operations, respectively. The computational formula is as follows:

(8) $$F_{a}^{1}=GA(F_{d}),F_{m}^{1}=GM(Fd)$$

To maintain the correlation of neighboring sample points, and given that larger sizes increase the computational cost, $F_{a}^{1}$ and $F_{m}^{1}$ are downsampled to $F_{a}^{2}\in R^{\frac{W}{2}\times \frac{H}{2}\times 1}$ and $F_{m}^{2}\in R^{\frac{W}{2}\times \frac{H}{2}\times 1}$, i.e.,:

(9) $$F_{a}^{2},F_{m}^{2}=Down\left(F_{a}^{1},F_{m}^{1}\right)$$

To emphasize the characteristics of the crack image (e.g., greyscale, texture, etc.) and ascertain the most contributing feature-space location, a dynamic modulation coefficient γ is introduced between $F_{a}^{2}$ and $F_{m}^{2}$. This coefficient is dynamically adjusted to accentuate the texture characteristics of the crack when the model is obtained, thus facilitating the model’s identification of the crack. Concurrently, it enhances the feature signals related to the crack, thereby ensuring a comprehensive capture of the crack characteristics. The Dynamic is used to denote the dynamic modulation coefficient generator.

(10) $$F_{a}^{3}=Dynamic\left(F_{m}^{2},\gamma \right)\times F_{a}^{2}$$

(11) $$F_{m}^{3}=Dynamic\left(F_{a}^{2},\gamma \right)\times F_{m}^{2}$$

After that, $F_{a}^{3}$ and $F_{m}^{3}$ are upsampled to maintain the similarity of neighboring points, and the two are spliced into Conv and Sigmoid to generate the mask $F_{mask}\in R^{W\times H\times 1}$.

(12) $$F_{a}^{4},F_{m}^{4}=Up\left(F_{a}^{3},F_{m}^{3}\right)$$

(13) $$F_{mask}=Sigmoid\left(Conv\left(F_{a}^{4}+F_{m}^{4}\right)\right)$$

A substantial amount of redundant information is present in the image of the actual crack-detection scene. The mask obtained through the aforementioned operation is multiplied with the original feature $F_{d}$ to generate $F_{di}$, $F_{di}=F_{mask}\times F_{d}$. This process not only removes the channel redundancy but also specifies the feature block by modulation coefficients in the feature reconstruction, which further removes redundant information that is unfavorable to crack detection and improves the accuracy of the model detection.

## 3. Results

### 3.1. Experimental Setup

#### 3.1.1. Datasets

**Figure 9:**
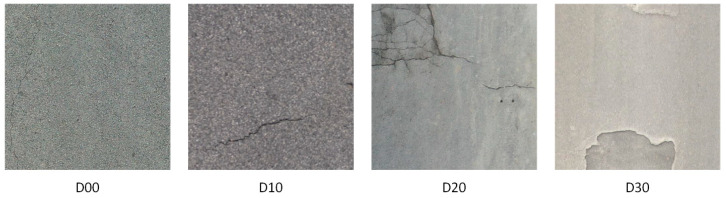
Visualization of road disease classification in the RDD2022 dataset.

The RDD2022 [39] dataset comprises 26,341 road images from China, India, Japan, and Norway, thus providing a comprehensive representation of a diverse array of environmental conditions and breakage categories. As shown in Figure 9, the dataset is annotated with approximately 65,000 instances of deterioration, which are classified into four categories: longitudinal crack (D00), transverse crack (D10), alligator crack (D20), and pothole (D30). The present study is principally concerned with the task of cement crack segmentation; as such, the cement data in the RDD2022 dataset are selected for research. Given the scarcity of pure cement crack data in the RDD2022 dataset, we retain some of the tarmac crack data with similar characteristics to the cement cracks in the data screening process, intending to ensure the diversity of the training data and enhance the model’s generalization ability.

**Figure 10:**
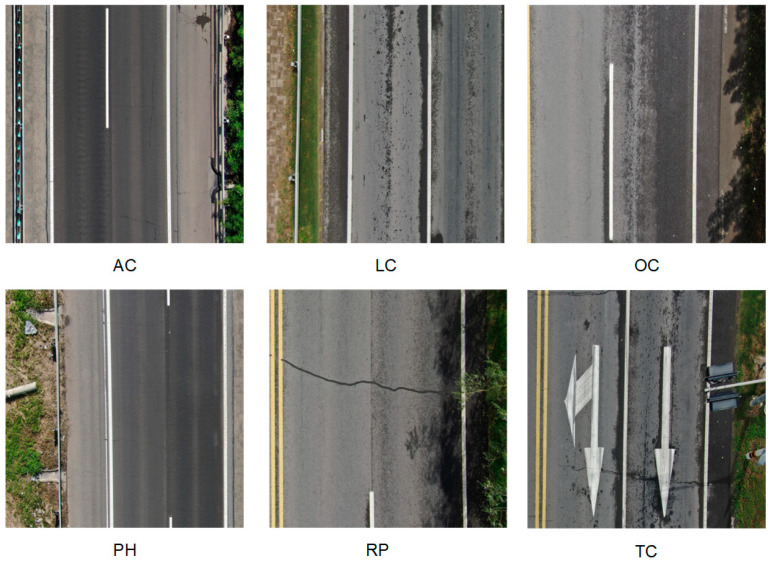
Visualization of road disease classification in the UAV-PDD2023 dataset.

The UAV-PDD2023 [40] dataset serves as a benchmark for road damage detection, leveraging drone technology to enable efficient and cost-effective infrastructure monitoring. Captured at a standardized flight altitude of 30 m, the dataset consists of 2440 high-quality images with 11,158 meticulously labeled instances following the PASCAL VOC annotation format, ensuring consistent resolution and perspective for reliable analysis. As shown in Figure 10, it provides precise annotations for six critical road damage categories—longitudinal cracks (LC), transverse cracks (TC), alligator cracks (AC), oblique cracks (OC), repair patches (RP), and potholes (PH)—offering comprehensive coverage of common deterioration patterns. The dataset is partitioned into training (70%), validation (20%), and test (10%) sets to facilitate robust model development and evaluation.

#### 3.1.2. Experimental Environment

The present experiment is conducted using the deep learning framework PyTorch. In terms of hardware, a high-performance NVIDIA GeForce RTX 3090 (Santa Clara, CA, USA) graphics card with 24 GB of video memory is utilized, which is capable of efficiently handling large-scale data computation. Concurrently, the 12th generation Intel Core i7-12700KF processor is selected, with a main frequency of 3.6 GHz, which supports multi-threaded parallel computing. In this study, the standard training settings commonly utilized in YOLO-based detectors were adopted, as these have been empirically validated to be stable across a range of benchmarks. The learning rate was set to 0.001 to achieve a balance between convergence speed and model stability. The batch size was configured to 64 to optimize the parallel computing capability of the GPU while preventing memory overflow. The IoU threshold was set to 0.7 to ensure higher localization accuracy in the target segmentation task. The number of epochs was set to 300 to ensure the model is fully trained and to prevent overfitting.

#### 3.1.3. Evaluation Indicators

1. Precision

Precision is defined as the proportion of samples predicted by the model to be in the positive category that are actually in the positive category. The formula is as follows:

(14) $$Precision=\frac{TP}{TP+FP}$$

TP (True Positive) denotes the number of correctly predicted positive class samples, while FP (False Positive) signifies the number of negative class samples erroneously predicted as positive.

2. Recall

The recall rate is defined as the proportion of all samples that are correctly identified as positive classes by the model. The formula is as follows:

(15) $$Recall=\frac{TP}{TP+FN}$$

where FN (False Negative) is the number of positive class samples that were incorrectly predicted as negative classes.

3. mAP (Mean Average Precision)

mAP is a widely employed evaluation metric in the field of image segmentation. It is calculated by determining the average precision AP for each category and subsequently averaging them. AP is indicative of the area under the precision–recall curve. A larger AP indicates a superior model detection performance. The calculation of AP is as follows:

(16) $$AP=\int_{0}^{1}Precision\left(Recall\right)$$

The specific mAP calculation can be performed using different IoU thresholds to evaluate the model’s performance. The following are the most common:

mAP@0.5: This is the mean Average Precision (mAP) when the intersection over union (IoU) threshold is 0.5. This means that the detection frame is correctly detected when the overlap between the detection frame and the real frame reaches 50%.

mAP@0.5:0.95: This is the average of the mAPs under multiple IoU thresholds (e.g., 0.5, 0.55, …, 0.95).

### 3.2. Backbone Comparison Experiment

To evaluate the performance of different backbone structures in crack-detection tasks, we conduct comparative experiments on YOLOv11n, Double-YOLOv11n (two SDBs), and Double-YOLOv11n-FDB (Dual-Domain Collaborative Feature Extraction Architecture, SDB+FDB) in the same experimental environment. The input resolution of all models was set to 640 × 640, and the experimental results are shown in Table 1.

The experimental findings demonstrate that YOLOv11n, serving as the base model, exhibits high detection accuracy in the crack-detection task (mAP50 attains 87.50%), yet there is considerable scope for enhancing its recall. The Double-YOLOv11n model employs a double-backbone structure, leading to enhancements in mAP50 and mAP50-95 to 87.70% and 59.90%, respectively, and an improvement in recall to 59.90%. The experimental findings demonstrate that the double-backbone configuration enhances the feature extraction capability and improves the detection rate of crack targets. The mAP50 and mAP50-95 are 59.90% and 82.50%, respectively. These results indicate that the double-backbone structure enhances the feature extraction capability and improves the detection rate of crack targets. However, precision exhibits a slight decrease to 85.10%, which may be attributable to the feature redundancy introduced by the double-backbone structure, thereby affecting the accuracy of detection. The integration of FDB within the framework of Double-YOLOv11n has been shown to enhance mAP50 and mAP50-95 to 88.30% and 60.50%, respectively, while concomitantly achieving a substantial enhancement in precision to 88.40%. This outcome suggests that the FDB configuration offers notable advantages in crack-detection tasks. The FDB enhances the representation of crack edges through frequency-domain information extraction, enabling the model to distinguish cracks from complex backgrounds more efficiently, thus reducing false detections and improving detection accuracy. The integration of the Double-YOLOv11n architecture, as devised by double backbone, serves to enhance the model’s overall feature extraction capability. The incorporation of the Double-YOLOv11n-FDB further optimizes the accuracy of crack detection through the introduction of frequency-domain information, thereby ensuring the model’s superior performance in several key indicators. The experimental findings demonstrate that the FDB structure can effectively enhance the expression of crack-edge features and improve the robustness of detection, especially in complex backgrounds and low-contrast crack-detection scenarios.

### 3.3. Detection Head Comparison Experiment

In the task of crack detection, the design of the detection head exerts a direct influence on the model’s localization accuracy and classification performance for crack targets. To this end, the present study involves a series of comparative experiments on different detection head structures, namely YOLOv11n (basic detection head), YOLOv11n-DSConv (Dynamic Snake Convolution [41]), YOLOv11n-DCNv4 (Deformable Convolution v4 [42]), and YOLOv11n-DIA-Head. All experiments were conducted under the same environment and hyperparameter settings, and the results are shown in Table 2.

The experimental findings demonstrate that YOLOv11n-DSConv employs DSConv, resulting in a mAP50 of 88.00% and a mAP50-95 of 60.10%. This enhancement signifies an augmented capacity for crack adaptation. However, the recall drops to 80.50%, indicating that some crack areas may not be fully detected. YOLOv11n-DCNv4 adopts DCNv4, which improves the spatial deformation ability of the model and enables it to adapt to the irregular morphology of cracks more flexibly. While the recall increased to 81.60%, the mAP50 and mAP50-95 only witnessed marginal improvement, at 87.80% and 59.60%, respectively. This finding suggests that, while deformable convolution facilitates adaptation to crack morphology, its overall detection accuracy remains constrained. In contrast, YOLOv11n-DIA-Head enhances the extraction of crack-detection features based on the Dynamic Head structure by introducing the DIA Module. The method achieves 88.20% and 60.10% on both mAP50 and mAP50-95, respectively, while maintaining a precision of 87.60%, thus demonstrating superior overall performance. The experimental results demonstrate that the DIA Head effectively suppresses the interference of background noise through the feature screening and dynamic enhancement mechanisms, and reduces the redundant information in the crack target-extraction process, which makes the detection results more accurate.

**Figure 11:**
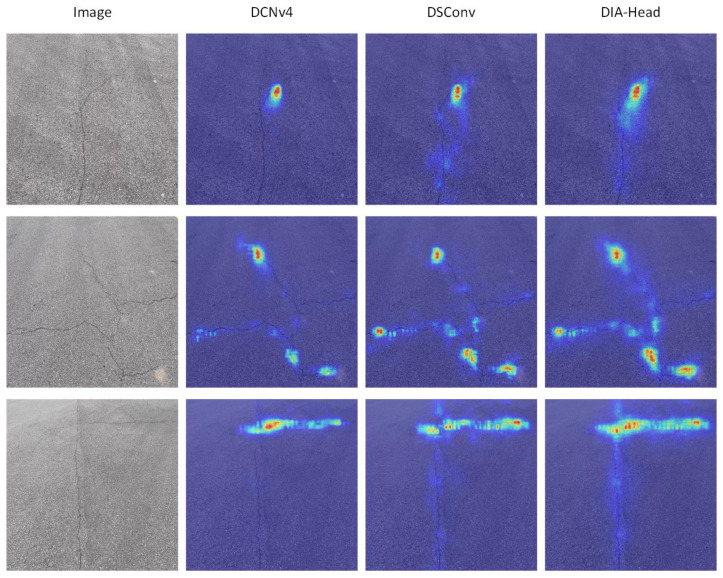
Comparison results of thermogram visualization of three different detection heads.

As illustrated in Figure 11, the detection and localization capabilities of three distinct detection heads (DCNv4, DSConv, and the DIA Head) are demonstrated for cracks in the crack-detection task, utilizing heat maps. Each row corresponds to a different crack scenario, and each column shows the original image, DCNv4, DSConv, and the DIA Head from left to right. It is evident that DCNv4 demonstrates a certain degree of detection capability for crack regions and can localize some significant crack regions. However, it falls short of capturing the continuity of the overall crack shape. Conversely, DSConv has enhanced the localization range and shape-fitting capabilities, resulting in a more comprehensive coverage of the crack structure. However, this method is susceptible to background interference. In contrast, the DIA Head is more focused and coherent in crack localization, and more accurate in feature extraction in crack regions, while effectively suppressing the false response in non-crack regions. This reflects its robustness in complex backgrounds and sensitivity to fine cracks. These visualization results further validate the advantages of DIA Head in improving the accuracy and stability of crack detection.

### 3.4. Complex Scenarios: Crack Detection Under Challenging Conditions

**Figure 12:**
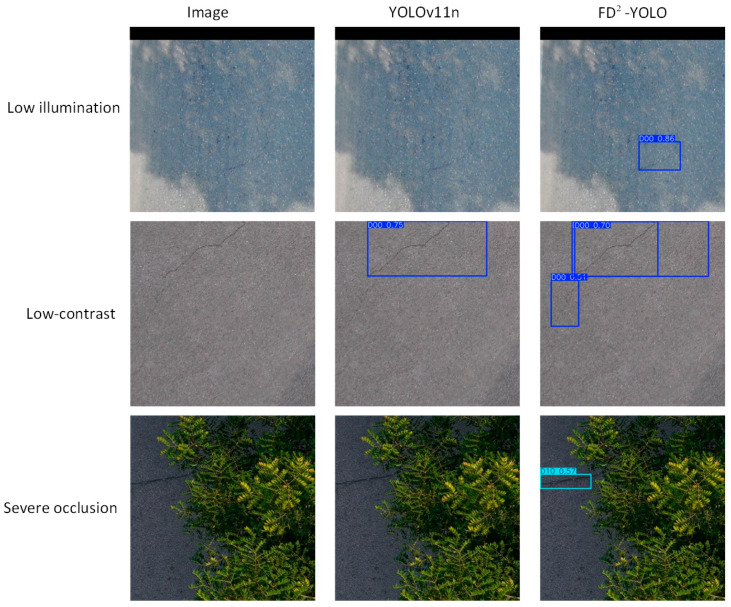
Crack-detection comparison between YOLOv11n and FD^2^-YOLO under challenging conditions.

To verify the robustness of the proposed FD^2^-YOLO model in real-world applications, experiments were conducted under various conditions, including low illumination, severe occlusion, and low-contrast environments. It is a fact that such conditions are common in practical crack-detection tasks. As demonstrated in Figure 12, the proposed FD^2^-YOLO exhibits a substantially superior visual performance in comparison to the baseline model. In conditions of low luminosity, FD^2^-YOLO successfully detects barely discernible crack features, a capability facilitated by its enhanced frequency-domain representation and dynamic feature fusion mechanism. In circumstances of severe occlusion, the model evinces a noteworthy aptitude for predicting fractures, even in scenarios where particular regions of the target are obscured. In scenes exhibiting low contrast, wherein the cracks demonstrate minimal intensity disparity relative to the surrounding background, the FD^2^-YOLO model consistently generates discernible detection outcomes, a capability that the baseline model conspicuously lacks. It is evident from the observations that FD^2^-YOLO is capable of maintaining high levels of detection performance in challenging conditions. This finding serves to further substantiate the claimed robustness and practical adaptability of the method.

### 3.5. Ablation Experiment

To verify the effectiveness of the Dual-Domain Collaborative Feature Extraction Architecture, the DIFF, and the DIA Head, we conduct systematic ablation experiments on the YOLOv11n benchmark model. As demonstrated in Table 3, the incorporation of all modules substantially enhances the performance of the crack-detection task. Specifically, the mAP50 of the full model FD^2^-YOLO reaches 88.80%, which is 1.3 percentage points higher than that of the benchmark model (87.50%). This outcome validates the rationality of the module design.

The Dual-Domain Collaborative Feature Extraction Architecture employs the SDA+FDA dual backbone network to extract spatial- and frequency-domain features, with the wavelet transform being utilized to enhance the detection of crack elongated edges and local discontinuous regions. This results in an improvement of 0.8% (88.30%) in mAP50 compared to the base model YOLOv11n. The DIFF module fuses the spatial- and frequency-domain features of the dual backbone network through a dynamic weight learning mechanism, showing an improvement of 0.3% (88.60%) compared to Double-YOLOv11n-FDB, which demonstrates its ability to exploit cross-domain feature complementarities. The DIA Head employs a deformable mask to effectively suppress background interference, achieving a mAP50 of 87.70% in stand-alone operation. The primary benefit of the DIA Head is that it employs a geometric adaptive mechanism to enhance the sensitivity of the detection head to the crack shape. Furthermore, when employed in conjunction with the Double-YOLOv11n-FDB-DIA-Head Collaborative Feature Extraction Architecture, the performance of the DIA Head is enhanced to 88.50%. The integration of the Dual-Domain Collaborative Feature Extraction Architecture, DIFF, and DIA-Head modules (FD^2^-YOLO) has been shown to enhance model performance, with an increase in mAP50 to 88.80%, representing a substantial improvement of 1.3 percentage points compared to the benchmark model (87.50%). The experimental findings demonstrate that the synergistic optimization mechanism among the modules effectively addresses the multidimensional challenges inherent in the complex crack-detection task.

### 3.6. Comparison Experiment

The FD^2^-YOLO proposed in this paper demonstrates significant advantages in the crack-detection task. As demonstrated in Table 4, under the input resolution of 640 × 640, FD^2^-YOLO exhibits superior performance in comparison to the prevalent detection model, achieving a mAP50 of 88.80% and a recall of 82.50%. This enhancement of 0.8 percentage points over the current optimal YOLOv12n (mAP50: 88.00%), and an improvement of 0.8 percentage points over YOLOv8n (recall: 81.70%) and YOLOv12n (recall: 80.50%) by 0.8% and 2.0%, respectively. The enhancement in performance can be attributed to the following factors: firstly, the FDB module has been enhanced to extract high-frequency edge features more effectively; secondly, the DIFF module has been developed to facilitate cross-domain dynamic fusion; and thirdly, the DIA Head has been equipped with a geometric adaptive mechanism to effectively solve the blurring problem of the localization of elongated cracks in complex backgrounds. A comparison of FD^2^-YOLO with the RT-DETR series model of transformer architecture reveals that the former has more significant advantages, with its mAP50 improved by 3.8% compared with RT-DETR-L (85.00%), and the recall gap widened to 3.6% (82.50% vs. 78.90%). The experimental findings demonstrate that FD^2^-YOLO substantially enhances the modeling capacity of crack morphological diversity (e.g., bending, bifurcation) through the integration of spatial–frequency-domain modeling and end-to-end optimization mechanisms. This approach addresses the requirement for crack detection in complex backgrounds. Although this improvement comes with a moderate increase in parameter count (5.9M), FD^2^-YOLO still maintains a lightweight architecture while significantly boosting accuracy and recall, striking a practical balance between efficiency and performance.

**Figure 13:**
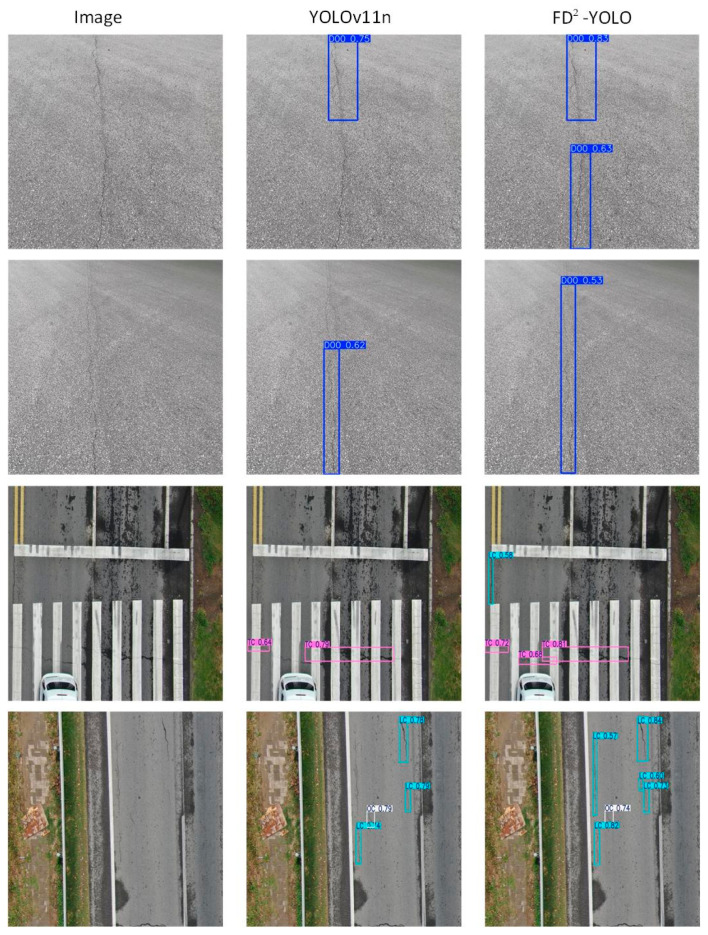
Performance comparison between FD^2^-YOLO and YOLOv11n on RDD2022 dataset.

The visualization of the detection results presented in Figure 13 further corroborates the aforementioned conclusions. The base model YOLOv11n demonstrates clear detection incompleteness or misdetection in several standard scenarios, which makes it challenging to meet the demand for high-precision detection. Conversely, FD^2^-YOLO exhibits superior performance in accurately encompassing the crack area with a reduced number of false positives, while also successfully identifying and localizing complex crack structures with precision.

To further validate the generalization performance of FD^2^-YOLO, additional experiments were conducted on the UAV-PDD2023 dataset, which contains more diverse backgrounds and finer-grained pavement crack details. As shown in Table 5, FD^2^-YOLO consistently outperforms the baseline models, achieving the highest mAP50 of 67.90% and mAP50-95 of 35.90%, with a recall of 61.20% and a precision of 75.90%. Compared to the best-performing baseline YOLOv11n (mAP50: 66.70%, recall: 61.90%), FD^2^-YOLO improves mAP50 by 1.2 percentage points and precision by 3.6 percentage points, while maintaining comparable recall. Notably, FD^2^-YOLO significantly surpasses YOLOv12n and YOLOv8n, with a mAP50 gain of 8.7% and 3.2%, respectively. Compared to transformer-based RT-DETR-L, FD^2^-YOLO demonstrates a substantial advantage with an 18.0% improvement in mAP50 and an 11.4% increase in recall. These results reinforce the robustness and effectiveness of FD^2^-YOLO in handling complex aerial imagery scenarios with small-scale and morphologically varied cracks.

### 3.7. Training Stability Analysis

**Figure 14:**
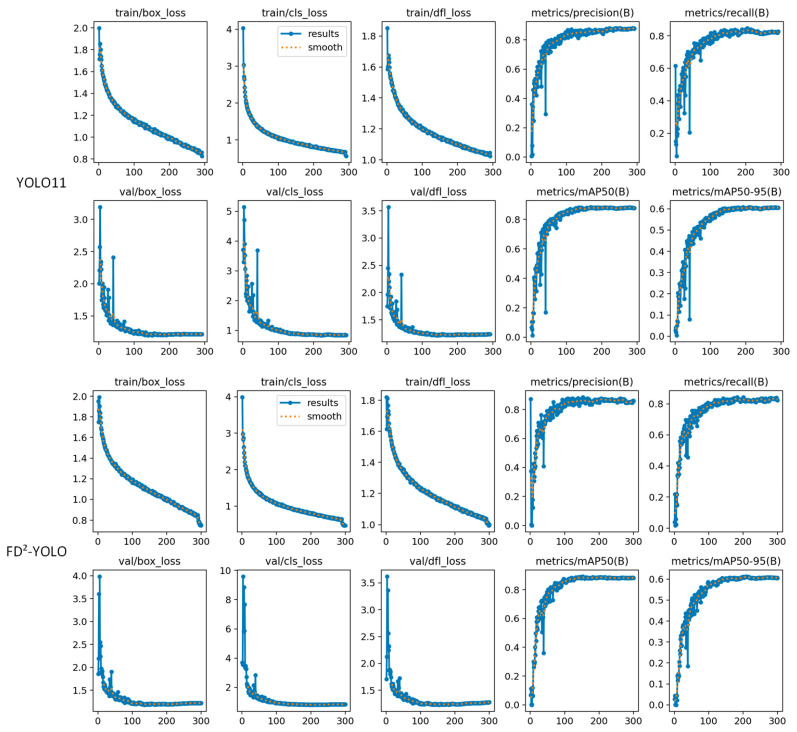
Comparative training dynamics of YOLOv11 and FD^2^-YOLO on RDD2022 dataset: loss convergence and metric evolution curves.

This study aimed to assess the training stability and convergence behavior of the proposed FD^2^-YOLO model. To this end, key performance metrics were recorded over the training process, including training loss, validation loss, precision, recall, and mean Average Precision (mAP). As demonstrated in Figure 14, FD^2^-YOLO exhibits a stable and consistent training trajectory. The training loss demonstrates a consistent decrease across epochs, while the validation loss remains relatively stable, suggesting effective generalization and the absence of overfitting. The curves for precision, recall, and mAP demonstrate a progressive and monotonic enhancement, reflecting the model’s robust feature learning capability and optimization robustness.

In comparison, the baseline YOLOv11 model demonstrates more volatile trends. The training loss fluctuates notably in the early epochs, and the validation loss curve is less stable, suggesting reduced training efficiency and susceptibility to suboptimal convergence. In addition, the mAP of YOLOv11 increases more gradually and eventually reaches a lower threshold than that of FD^2^-YOLO. The discrepancy highlighted serves to emphasize the advantages of the dual-domain design and dynamic fusion strategy that have been employed in FD^2^-YOLO. The combination of these two elements serves to enhance representation learning and ensure more stable metric growth.

The training curves demonstrate that FD^2^-YOLO exhibits superior performance and higher training stability and convergence efficiency in comparison with YOLOv11. This validates the effectiveness of the proposed architectural enhancements.

## 4. Discussion

The experimental findings presented in this study demonstrate the efficacy of the proposed FD^2^-YOLO model in detecting cement cracks under a range of challenging conditions. FD^2^-YOLO consistently outperforms existing models, including YOLOv11n, YOLOv8n, and RT-DETR variants, in terms of mAP50, recall, and false detection rate. On the RDD2022 dataset, FD^2^-YOLO achieves a mAP50 of 88.8%, representing improvements of 1.3% and 1.6% over YOLOv11n (87.5%) and YOLOv8n (87.2%), respectively. It also achieves a recall of 82.5%, surpassing YOLOv11n and RT-DETR-L by 1.8% and 3.6%. In terms of precision, FD^2^-YOLO reaches 87.8%, effectively reducing false detections compared to baseline models. To further evaluate generalization capability, FD^2^-YOLO was tested on the UAV-PDD2023 dataset, where it achieved a mAP50 of 67.9% and a recall of 61.2%, outperforming YOLOv11n (66.7%, 61.9%) and showing substantial gains over transformer-based RT-DETR-L (49.9%, 49.8%). These results quantitatively validate the effectiveness of integrating spatial- and frequency-domain representations, as well as the benefits of dynamic feature fusion and deformable attention mechanisms in enhancing feature learning and detection robustness across diverse real-world conditions.

The Dual-Domain Collaborative Feature Extraction Architecture plays a critical role in addressing the limitations of traditional single-stream convolutional networks. Previous studies have shown that spatial-domain networks alone are often inadequate in capturing fine-grained features such as hairline cracks and low-contrast textures. By introducing the frequency-domain branch and leveraging wavelet decomposition via the C3K2-LW module, the model can retain high-frequency details crucial for crack-edge and texture detection. This aligns with and extends earlier findings on wavelet-based feature enhancement in defect detection tasks.

Moreover, the DIFF module provides an effective strategy for aligning heterogeneous feature distributions from different domains. The Hadamard product-based attention mechanism enhances the interpretability and adaptability of the fused features, enabling the network to better distinguish cracks from complex backgrounds. These observations corroborate prior research on attention-guided fusion.

The DIA Head further contributes to the model’s performance by enabling adaptive focus on key features such as irregular morphology, grayscale variation, and localized deformation. While deformable convolution has previously been applied in segmentation tasks, our DIA Head demonstrates its effectiveness in detection scenarios.

Despite its strong performance, the model’s robustness under challenging conditions, such as severe occlusion and low illumination, remains to be fully explored. Future work may focus on integrating transformer-based modules into the dual-domain architecture to enhance long-range dependency modeling and further improve detection accuracy in complex environments.

## 5. Conclusions

In this study, we propose FD^2^-YOLO, a novel dual-stream network model. The model incorporates three key components: firstly, a Dual-Domain Collaborative Feature Extraction Architecture that jointly leverages spatial- and frequency-domain information to enhance the representation of crack edges and fine-grained structures; secondly, a DIFF module that effectively bridges the distribution gap between heterogeneous features and enables adaptive cross-domain integration; and thirdly, a DIA Head that improves the detection of slender, bifurcated, and low-contrast cracks through dynamic modulation and multi-scale contextual reasoning. The collective impact of these innovations is to address significant limitations in current research. This is achieved by enabling effective spatial–frequency synergy under complex conditions. In subsequent studies, the enhancement of the model’s generalization capabilities is intended through the incorporation of a more diverse range of datasets, encompassing a variety of materials and environmental contexts. Moreover, the integration of transformer-based modules will be investigated to enhance long-range dependency modeling and pixel-level localization. This will facilitate the development of more accurate and interpretable crack-detection systems in real-world infrastructure inspection scenarios.

## Figures and Tables

**Table 1:** Performance comparison of different backbones for crack detection on the RDD2022 dataset.

Model	Size	mAP50/%	mAP50-95/%	Recall/%	Precision/%
YOLOv11n	640 × 640	87.50	59.20	80.70	87.30
Double-YOLOv11n	640 × 640	87.70	59.90	82.50	85.10
Double-YOLOv11n-FDB	640 × 640	88.30	60.50	80.20	88.40

**Table 2:** Performance comparison of different sensor heads for crack detection on the RDD2022 dataset.

Model	Size	mAP50/%	mAP50-95/%	Recall/%	Precision/%
YOLOv11n	640 × 640	87.50	59.20	80.70	87.30
YOLOv11n-DSConv	640 × 640	88.00	60.10	80.50	87.60
YOLOv11n-DCNv4	640 × 640	87.80	59.60	81.60	87.10
YOLOv11n-DIA-Head	640 × 640	88.20	60.10	80.60	87.60

**Table 3:** Ablation study of key modules for crack detection on the RDD2022 dataset.

Model	FDB	DIFF	DIA Head	mAP50/%
YOLOv11n				87.50
Double-YOLOv11n-FDB	√			88.30
YOLOv11n-DIA-Head			√	87.90
Double-YOLOv11n-FDB-DIFF	√	√		88.60
Double-YOLOv11n-FDB- DIA-Head	√		√	88.50
FD^2^-YOLO	√	√	√	88.80

**Table 4:** Performance comparison of state-of-the-art models for crack detection (RDD2022 dataset).

Model	Size	mAP50/%	mAP50-95/%	Recall/%	Precision/%	Parameters/M
YOLOv11n [36]	640 × 640	87.50	59.20	80.70	87.30	2.6
YOLOv12n [43]	640 × 640	88.00	60.10	80.50	87.60	2.6
YOLOv8n [44]	640 × 640	87.20	59.20	81.70	85.40	3
YOLOv5n	640 × 640	86.70	58.40	80.20	84.50	2.5
RT-DETR-Resnet50 [45]	640 × 640	84.00	54.70	78.90	80.90	41.9
RT-DETR-l [45]	640 × 640	85.00	56.70	78.90	86.00	32
EfficientDet [46]	640 × 640	81.30	-	-	-	17
FD^2^-YOLO	640 × 640	88.80	60.30	82.50	87.80	5.9

**Table 5:** Performance comparison of state-of-the-art models for crack detection (UAV-PDD2023 dataset).

Model	Size	mAP50/%	mAP50-95/%	Recall/%	Precision/%	Parameters/M
YOLOv11n [36]	640 × 640	66.70	34.10	61.90	72.30	2.6
YOLOv12n [43]	640 × 640	59.20	28.90	53.70	67.30	2.6
YOLOv8n [44]	640 × 640	64.70	32.70	58.10	73.20	3
YOLOv5n	640 × 640	65.40	32.60	56.90	75.10	2.5
RT-DETR-Resnet50 [45]	640 × 640	58.40	29.00	56.80	66.50	41.9
RT-DETR-l [45]	640 × 640	49.90	23.10	49.80	58.70	32
EfficientDet [46]	640 × 640	46.60	-	-	-	17
FD^2^-YOLO	640 × 640	67.90	35.90	61.20	75.90	5.9

## Data Availability

The dataset under consideration is derived from RDD2022, which can be accessed via the following link: https://github.com/sekilab/RoadDamageDetector (accessed on 26 May 2025).

## Abbreviations

The following abbreviations are used in this manuscript:

Abbreviation	Full name
CBS	Convolution–BatchNorm–Silu
DBS	Depthwise Separable Convolution–BatchNorm–Silu
DIFF	Dynamic Inter-Domain Feature Fusion Module
DIA-Module	Deformable Interactive Attention Module
SDB	Spatial-Domain Backbone
FDB	Frequency-Domain Backbone
LWN	Learnable Discrete Wavelet Transform Node
DIA-Head	Deformable Interactive Attention Detection Head
